# Determinants of Satisfaction with Solid Waste Management Services: A Central–Local Comparison in China

**DOI:** 10.3390/ijerph19084610

**Published:** 2022-04-11

**Authors:** Aiqin Wang, Xuyang Chen, Xu Wang, Jia Wei, Liying Song

**Affiliations:** School of Economics and Finance, Xi’an Jiaotong University, Xi’an 710061, China; 3120319106@stu.xjtu.edu.cn (X.C.); wx20001128@stu.xjtu.edu.cn (X.W.); jiawei0626@xjtu.edu.cn (J.W.); songly@mail.xjtu.edu.cn (L.S.)

**Keywords:** solid waste management services, satisfaction, central–local comparison, China

## Abstract

The Chinese central government proclaimed a mandatory or demonstration waste separation policy in some cities or counties to manage the increasing amounts of generated waste since 2017. Many cities and regions have also begun to build waste separation management systems and institutions, and community managers have created waste management rules and provided separation management services under the guidance of local government. However, little is known about how these policies or services have performed and the degree of residents’ satisfaction, especially regarding the central government. Therefore, the purpose of this study was to analyze the consequence of these policies using residents’ community and national satisfaction levels with solid waste management services (SWMS) and their determinants. An online survey in 2021 showed that the overall satisfaction levels of communities and national SWMS are similar, but the differences between rural and urban regions are significant. Residents’ satisfaction with community SWMS in urban regions was higher than national satisfaction, and the results in rural regions was contrary. The determinants of community and national satisfaction were also different and varied by region. To improve satisfaction, the government need to standardize basic management systems in different types of regions, gradually improve management services and institutions in rural areas and non-pilot cities and provide special services or facilities for less healthy residents.

## 1. Introduction

With the rapid development of society and the economy in China, the domestic waste volume has increased rapidly—China became the largest municipal waste generation country in the world, outperforming the United States, in 2004 [1,2]. The domestic waste volume in 2019 reached 242 million tons, and there is continuing damage to the ambient environment and residents’ health because of the lack of separated waste collection management services systems [3,4,5,6]. To solve the waste problem involving the lives of thousands of families, President Xi Jinping encouraged residents to adopt a solid waste separation habit in December 2016, and waste separation collections were again on the agenda. The central government announced that 46 cities would have to create solid waste mandatory separation plans in 2017 [7]. Shanghai, which is the largest megacity in China, has taken the lead in establishing a municipal solid waste separation and recycling system, but questions about inadequate separate collection vehicles and limited waste treatment capacity still exist [8,9,10]. At same time, 100 counties began to be created the rural solid waste separation demonstration counties, but the rate of sorting waste was still relatively low, and separation bins were lacking [11,12]. Then, in June 2019, all prefecture-level and higher cities began to carry out a waste separation plan, but a small number of local communities were only chosen as the pilot communities for separating waste [13]. Though these policies have some problems or shortages in the implementation process, they present a model or experience for other non-pilot cities and rural regions to create waste management systems [10,11,12,14,15].

Different levels of local government have carried out this policy based on the residents’ participating willingness and community facilities, while the central government enacted a mandatory waste separation policy or waste demonstration programs; thus, residents may have different perceptions about the waste services or policies of central and local government [11,15,16]. The public showed more positive sentiments regarding a flexible waste separation policy than mandatory waste separation policy [15]. The satisfaction with solid waste management services (SWMS) is an effective indicator to evaluate the return or performance of the services provided with consumer satisfaction theories [17,18,19,20,21] and allows a comprehensive evaluation of service efficiency, responsiveness, equity and effectiveness under limited human, material and financial resources [22,23], which can provide feedback and development directions for policy makers and implementers to improve policy and implementation approaches [24]. Some studies of other countries—for example, Malaysia [25], Spain [26,27], Kenya [28], India [29], Slovakia [30] and Italy [31]—also evidenced that satisfaction varies with the situation and quality of SWMS in the areas and countries and demonstrated that 30–84% residents were satisfied with the community SWMS [25,26,27,28,29,30,31,32,33,34,35]. Satisfaction with SWMS will also accelerate residents’ separation behavior, services expectations and perceived quality and social interactions among communities [25,26,36,37,38]; then, it affects community social and environmental sustainability [39,40,41,42]. Under the mandatory waste separation policy from the central government and resilient communities’ waste management execution, it is necessary to determine whether residents have higher satisfaction with central government or local government or community managers. Hence, it is important to learn the status quo of satisfaction with community and national SWMS in China and compare the difference for effective environmental governance in the future.

Many studies focus on residents’ satisfaction with SWMS from local government in rural or urban regions before a mandatory or demonstration policy, but there is a lack of evaluations about the satisfaction with national policy or services and the central–local and urban–rural comparison [43,44,45,46,47,48,49,50,51,52]. In China, many studies have analyzed rural residents’ satisfaction in specific regions—for example, Jiangsu, Beijing, Hunan and Hangzhou Province—and showed that the level of satisfaction is 77%, 67%, 65% and 58%, respectively [43,44,45,46]. Only a few studies have analyzed satisfaction with SWMS using national data; for example, Li et al. showed that 85% of households were satisfied with waste collection facilities provided by local government using the data from 1450 households of seven provinces in rural China [47]. Other research works showed urban residents’ satisfaction with municipal solid waste management services—for example, the detailed non-pilot city of Tongling, some pilot cities of Harbin and Xining and all pilot cities—and found that 34%, 44%, 17% and 20% residents were satisfied with SWMS, respectively [48,49,50,51]. There are a few studies that have analyzed satisfaction with SWMS using nationwide data; for example, Wang et al. showed that 65% of residents were satisfied with waste separation bins using data from 672 respondents of 31 provinces in urban China [38]. These results were verified with different samples, questions and times, and the satisfaction with SWMS in urban regions is basically lower than rural regions [53].

There were some research works about the differentiation of hierarchical satisfaction about public services or environmental services that include SWMS [54,55,56,57], but few research works have compared the differences of central–local satisfaction levels with SWMS and their determinants. Studies showed that the satisfaction with public services from the central government was higher than local government in general, but different types of public services may have different satisfaction levels [54,55,56,57]. The main reason is the disparity of roles or responsibilities between central and local government, especially in China, and the differences of distances between the government and the people [58,59,60,61,62,63]. Residents in China think that central government is authoritative, which is the subject of making and advertising policy and supervising its implementation [54,55,64,65,66]. However, regarding the qualities of public services, residents often blame local government for failings for not meeting their demands [54,55,66,67,68]. These analyses were usually comprehensive, and it is not possible to give some detailed suggestion or direction about providing SWMS for central and local government.

The factors that impacted satisfaction when applied to solid waste separation collection, management or treatment services in the previous studies include many items [25,26,27,28,29,30,31,32,33,34,35,36,37,38,39,40,43,44,45,46,47,48,49,50,51,52,53,54,55,56,57]. Some previous studies have used the structural equation model, service quality (SERVQUAL) model, expectancy disconfirmation model (EDM) or descriptive and factor analysis methods to evaluate the determinants of satisfaction with SWMS or public services [26,27,28,29,30,31,32,33,34,35,39,40,43,44,45,48,49,50,51,52,53,69,70], but there has been little research on the important factors and their effect degree that can be used to synthetically evaluate the performance of services from the structures and management processes of SWMS [25,26,27,38,46,47,52]. Considering these results together, we can broadly classify these factors into four categories. First, individual socioeconomic characteristics [25,43,44,45,51]–income and education are the main factors reducing satisfaction with SWMS [25,26]. Older residents have higher satisfaction, as the community environment and services were developed and improved to a great context over their lifetimes. In addition to these factors, types of house and occupation also increase satisfaction [25,43]. Second, environmental knowledge or perceptions impact satisfaction—including environmental participation, perceived value of sorting and cognitive traits in detail [26,27,38,39,71]. With the interaction with other residents, environmental activities can increase environmental perceptions and thus increase satisfaction [45]. The cognitive traits concerning waste management include familiarity with laws and regulations. Third, waste management facilities, completeness of services and service quality or frequency also impact the satisfaction with local government [26,27,38,39,43,44,45,46,47]—investing in environmental infrastructure or waste sorting management facilities in the communities can greatly enhance levels of satisfaction [8,26,27,44]. The reasonable arrangement of waste separation facilities and collection methods also affect satisfaction with waste management [8]. The neatness of waste collection crew, the reliability of waste collection and service quality (including the frequency of waste cleanliness and collection and their pollution impact in the process of transportation and disposal) all impact satisfaction [16,25,43,44,45]. The technological innovation in the waste disposal process, such as the pyrolysis conversion of polymer wastes to noble fuels, the usage of an automatic mode in the waste treatment and the biomass combustion of waste composed if wood material, was a valid method to increase the efficiency of waste disposal and improve the waste-to-energy conversion, thus increasing residents’ satisfaction [72,73,74]. Fourth, waste-related institutions that produce policies such as community rules or regulations about waste and waste charge methods and fees affect satisfaction [45,46,47,48]. The charging standard for management and treatment directly determined the quantity and level of service, so residents’ payments for services enhanced their satisfaction [45,49,57,75,76].

Based on the literature review above, the following hypotheses are proposed: H1. Satisfaction with national SWMS is higher than community SWMS, and the difference is not large among regions and cities. H2. Residents’ socioeconomics characteristics have a significant impact on community and national satisfaction level. Income and education have a negative impact on community and national satisfaction levels. Age has a positive impact on community and national satisfaction levels. H3. Environmental perceptions have a positive impact on central-local satisfaction. H4. The situation of solid waste management has a positive impact on community satisfaction and no impact on national satisfaction. H5. There is a significantly positive correlation between waste institutions and community and national satisfaction.

This study is the first paper using the country-wide data to explain and compare the difference of central–local satisfaction using the ratings of national and community satisfaction with SWMS and analyze the determinants in China. This can creatively give researchers and service providers a general view about the effect of services and institutions on community and national satisfaction and the implementation pathway of waste separation policy. This paper has three specific aims: (1) to describe the differences between community and national satisfaction levels, especially in rural and urban regions and non-pilot and pilot cities; (2) to describe and compare the relationship between solid waste management services and institutions and the community and national satisfaction levels with SWMS in different regions; (3) to analyze the factors that determine satisfaction with SWMS at the community and national level. The structure of the paper is as follows. Section 2 presents the method of data collection and the models used. Section 3 presents the results, which include the description result, the relationship between solid waste management services and institutions and satisfaction with community and national SWMS and the determinants of satisfaction. Section 4 is the discussion, which discusses the results and the implications, compares the result with precious studies, explains the reason for the results and shows the limits of this work. Section 5 is the conclusion, which shows the overall results and makes suggestions for central and local government and the directions of future research.

## 2. Data and Methods

### 2.1. Data Collection

We designed questionnaires and conducted an online survey about SWMS using Jinshuju, which is an application program developed by ThoughtWorks. We then trained six undergraduates and two graduate students as the seed investigators to post the questionnaires. The participants needed to meet four basic conditions: (1) 18 years old and above; (2) they could finish the survey using Wechat; (3) they could only submit the answers through the same IP address once—any further attempts were restrained by Jinshuju; and (4) voluntary participation. Before the formal investigation, we carried out a pilot survey to test the questionnaire’s rationality and validity and improved the questions. For this, the seed investigators sent the survey questionnaires to recognized friends or relations individually or sent it to members of different groups using the method of group sending of Wechat. We did not publish the questionnaire in our circles of friends to avoid transmitting it optionally and to ensure the quality and availability of the data. After the survey, we randomly sent a small remuneration to the participants as an incentive to enhance the response rate for the questionnaires [77]. Ten percent of participants did not accept the incentive as they were perfectly willing to complete the survey and support the scientific research, so 90% of participants received the remuneration, which was on average USD 0.16 dollars (i.e., RMB 1 in China). Finally, 1200 participants filled out the questions between 4 and 10 February 2021, which was 1 week ahead of the spring festival and ensured that the information had the right context. Because of the missing socioeconomic variables, 1189 samples were usable, and they were from 185 cities in 31 provincial areas of China. Based on the residential districts of participants, the samples contained residents in the urban and rural regions. For the urban regions, we divided the cities into waste separation pilot cities and non-pilot cities based on the filled-in address and country documents. Though the samples of the survey are not representative of the overall situation, it can give the government in China and other developing countries some suggestions about SWMS. In the sample, 70% of residents lived in urban regions, and 49% of them lived in a pilot waste separation city.

In order to explore the diversity and determinants of satisfaction for the actual service providers—community managers and policy makers—and national governments with SWMS, we first used the satisfaction with community and national SWMS as the dependent variables. The questions were “are you satisfied with the situations of community SWMS?” and “are you satisfied with the situations or policy of national solid waste separation management services?”, and the answer scoring was based on a five-point Likert scale. Second, based on the literature in Section 1, we asked what the situation was in terms of community SWMS and basic institutional systems, environmental perceptions and socioeconomic characteristics. In order to unify the rural and urban regions, the community SWMS covered the four critical stages of the solid waste management process, which are waste separation facilities, waste separation collection behavior, waste transportation and waste disposal [1,3,5,9]. Waste separation facilities referred to the communities having three or four waste separation collection bins, which severally included dry waste, wet or kitchen waste, harmful waste bins, or recyclable waste, kitchen or household food or wet waste, hazardous waste and other waste bins based on the different city or regional policies and the national waste separation standard [78], which was noted as 1; otherwise, this was noted as 0. Waste separation behavior refers to residents’ personal or community sanitation workers’ separation collection behavior in the communities, which was noted as 1; a lack of such behavior was noted as 0. Waste disposal included four disposal methods: unregulated dumping, bury, burn and waste recycling and reuse (which is waste-to-energy, composting and centralized and separated waste disposal). To establish the comprehensive system of SWMS and encourage waste recycling and reuse, we re-coded waste disposal to waste recycling—that is, recycling and reuse, as 1; unregulated dumping, bury and burn was noted as 0.

Third, the institution systems included setting and enforcing waste separation rules, undertaking advertising programs and setting and receiving waste management fees at the community level. From the residents’ perspectives, the institutions should be effective when rules are enacted and advertised; then, the residents can obtain the information about SWMS and possibly engage in waste separation collection behavior. We also asked the participants about waste management fees at the community level. If the community had waste separation management rules, waste separation advertisement and waste management fees, this was coded as 1; otherwise, this was coded as 0.

Finally, we surveyed the environmental perceptions and the socioeconomic factors. The environmental perceptions included knowing about solid waste law and the pilot city list for waste separation and environmental activity. In addition, we collected socioeconomic characteristics data, which included gender, age, education, health and income. These variables and their definitions are also shown in Table 1.

### 2.2. Model and Methods

We used both descriptive and multivariate analyses to evaluate the determinants of community and national satisfaction with solid waste management services. Based on the sequential dependent variables, we constructed an Ologit (Ordered Logit) regression model to analyze the determinants of satisfaction with SWMS and compare the differences between community and national satisfaction. Some studies considered that the situation of SWMS in China is simply decided by the local government or community managers based on the community status and national policy, rather than being mainly affected by the residents [1,51,79,80,81], so we imagined that individual residents’ personal socioeconomic and environmental knowledge did not affect the community providing the SWMS. Because the relationship between the situation of SWMS and community institutions may be correlated, we used two steps to add the variables into the equations to compare and test the consistent impacts of the situations and institutions using the following equations:

(1) $${\mathrm{Sa}}_{i}\;=\;a\;+\;\beta_{1}{\mathrm{In}}_{i}\;+\;\alpha_{1}R_{i}\;+\;\alpha_{2}{\mathrm{En}}_{i}\;+\;\alpha_{3}X_{i}\;+\;\varepsilon_{i},$$

(2) $${\mathrm{Sa}}_{i}\;=\;a\;+\;\beta_{1}{\mathrm{In}}_{i}\;+\;\beta_{2}{\mathrm{Se}}_{i}\;+\;\alpha_{1}R_{i}\;+\;\alpha_{2}{\mathrm{En}}_{i}\;+\;\alpha_{3}X_{i}\;+\;\varepsilon_{i},$$

where Sa_i_ indicates the satisfaction level of resident i with SWMS, which includes satisfaction with community and national SWMS, and Se_i_ indicates whether the community where resident i lived provides SWMS (waste separation facilities and waste separation collection behavior, waste transportation and waste recycling). When a community provides SWMS, then Se_i_ is equal to 1; otherwise, it is equal to 0. In_i_ is the community institution, which includes waste separation rules, waste management fees and advertising. When a community has these institutions, then In_i_ is equal to 1; otherwise, it is equal to 0. R_i_ represents the region information of the communities, including region and pilot city, which are both dummy variables. In detail, region consists of the urban region, which is coded 1, and the rural region, which is coded 0; a pilot city is coded 1 and non-pilot city is coded 0. En_i_ is environmental perception, which includes environmental activity, knowledge about the environment law and the pilot city list. X_i_ is a vector describing the characteristics of a resident, including age, education, gender, health and monthly household income. Their definitions and values are shown in Table 1. The symbol α is constant term, and symbols β_1_, β_2_, α_1_, α_2_ and α_3_ are the coefficients to be estimated. ε_i_ is the error term. In addition, we use the Probit model to evaluate the impact on SWMS of all socioeconomic and environmental knowledge variables and community institutions in Appendix A Table A2. Furthermore, the Ordinary Least Squares (OLS) model and Oprobit model were added to our model to test for robustness, as shown in Appendix A Table A3.

### 2.3. Descriptive Statistics

The descriptive statistics for all variables are shown in Table 1. We found that more than half of the residents were women and undergraduates, and most of the sample comprised younger people, as the mean of age was 1.71. The health situation of most residents was good. The average monthly income of a family was USD 782-1564 dollar (i.e., 5000–10,000 RMB). Though environmental activity was low at only 30%, knowledge about environmental law and the waste separation pilot city list was relatively high at 74% and 86%, respectively.

Only 28% of residents reported that their communities provided waste separation collection facilities, and 62% of residents reported waste separation collection behaviors, so we found that most separation work is conducted by the waste separation workers. Because many residents, at about 443, did not know whether waste is transported, of all residents who knew this, 87% of the residents reported that the community had waste transportation services. Furthermore, waste recycling in 59% of communities was the main waste disposal method. In terms of community institutional actions, 64% of communities had seen advertisements that gave them waste-related knowledge, 56% of communities had rules about waste in the community, and 53% of communities collected a waste management fee.

In the sample, 70% of residents lived in urban regions and 49% lived in a pilot waste separation city. Overall, residents’ community and national satisfaction levels with SWMS were neutral, because the average values were 3.18 and 3.15, respectively.

## 3. Results

### 3.1. Solid Waste Management Services and Community Institutions in China

Concerning the situation of SWMS, we found that the overall supply level provided was lower in China than other countries according to the literature [6,14,42]. The SWMS in the urban regions were more comprehensive than those found in rural regions and non-pilot cities (Table 2), and all the waste services in urban regions were more developed than in rural regions with the support of government, showing the same patterns as precious research and social reality [4,79,80]. The gap in terms of waste facilities and behavior between cities is greater than between regions, but the gap concerning waste transportation and recycling between cities is lower than between regions. The largest and lowest disparities between the two region types were between waste separation facilities (17%) and waste transportation (5%). The ownership rate for separation facilities in urban regions was 33%, which was higher than rural regions, where it was 16%. Although there were fewer waste separation facilities in rural areas, separation behavior was relatively lower (56%) than in urban regions. Regarding the waste transportation services, a difference between regions existed, but none was found between cities. The rate of waste recycling is relatively high, and the gap between rural and urban regions is 9%.

We compared waste management institutional policies in urban and rural regions and found that there was no difference in the waste rules. However, the level of advertising promoting waste management was significantly different between rural and urban regions, but the gap was relatively minor. In fact, advertising about waste management in China was high, and this score was higher than that for waste rules. When services and institutional activities in different regions were compared, the largest divergence appeared for the waste management fee, which was only charged in 24% of communities in rural areas and was 42% lower than in urban regions. On the contrary, among cities, the largest difference was in waste advertisement and waste rules, and the lowest difference was found for the waste fee. This suggested that services and institutions in non-pilot cities are equally inadequate in rural areas and lower than in pilot cities.

### 3.2. Relationship between Solid Waste Management Services and Community Institutions and Residents’ Satisfaction in China

Overall, the satisfaction levels for community and national waste services are similar, as shown by the total sample in Table 3. From Table 3 and Appendix A Table A1, we found that the degree of rural residents’ satisfaction with the community SWMS, for which the average value is 3.06, was significantly lower than urban residents’ satisfaction, for which the value was 3.23, and the satisfaction in the non-pilot cities (3.19) was also lower than the pilot cities (3.27), though this was not significant. However, the residents in the urban regions were more satisfied with national services than rural residents (3.24 and 3.11, respectively), and there was no difference between non-pilot and pilot cities with regards to community and national service. In rural areas, the residents have higher satisfaction with national services than community services, but the residents in urban areas have higher satisfaction with community services than national services. Therefore, the satisfaction levels were inverted in rural areas, and there was no such phenomenon in urban areas.

In order to describe the relationship between SWMS and satisfaction in the rural and urban regions, we showed that the satisfaction level of residents in the community providing and not providing SWMS and institutions in Table 3. In addition, we also compare the differences in the relationships between them in non-pilot cities and pilot cities in Appendix A Table A1. In total, we found that, except for the waste fee, the residents were more satisfied with the communities and national government that provide SWMS services and have institutional activities. Only the waste management fee had a negative impact on the satisfaction of community and national SWMS, which is not in accordance with the previous studies [35].

Because there are different impacts on satisfaction between urban and rural regions, we analyze these regions separately. With the status of SWMS, we found that when residents reported that their community provided waste separation collection behavior and waste recycling services in rural areas, they expressed higher satisfaction with community SWMS than without these services. The satisfaction with national SWMS was mainly determined by the waste separation behavior. In urban regions, all SWMS had a positive impact on the satisfaction with community SWMS, but only waste transportation and waste recycling gave a similar result to the satisfaction with national SWMS. The reason may be that waste separation facilities and collection behavior are provided by the communities, but waste transportation and waste recycling are provided and invested in by the local or national government in urban areas. Whether in rural or urban regions, waste advertisement and waste rules both impacted the satisfaction with community and national SWMS. Only in urban regions did residents for whom no waste fee was charged in the communities reported that they had significantly higher satisfaction.

When we made a comparison between non-pilot and pilot cities, as shown in Appendix A Table A1, we showed that the results for the impact of waste separation facilities, collection behavior and waste recycling on community and national satisfaction levels with SWMS were the same as the results in urban regions. However, when residents reported that their communities provided waste transportation services, their national satisfaction in the non-pilot cities and community satisfaction in the pilot cities increased notably. The waste advertisements and waste rules also impacted the community and national satisfaction in the non-pilot cities. However, in the pilot cities, waste advertisement impacted the national satisfaction, the reason for which may be that advertisement activities give more information about the national policy and measurement, but waste rules impacted the community satisfaction, the reason for which may be that after obtain waste information through advertisements, waste rules give the resident local methods of separating or managing waste.

### 3.3. Determinants of Satisfaction with SWMS

Using the Ologit regression model, we analyzed the determinants of community and national satisfaction with SWMS from four aspects—socioeconomic characteristics, environmental perceptions, situation and institutions of SWMS—using the Ologit model in Table 4. Furthermore, we describe the result from the total, rural and urban samples to show the differences among them. In addition, we added the Oprobit and OLS regression in Appendix A Table A3 to robustly test the results and look for consistency in Table 4. We tested the relationship between all variables and the situation of SWMS and found that the relationship existed, and the result showed that the possible impact of individual residents’ socioeconomics characteristic on the situation almost barely existed, waste advertisement mainly affected the situation of SWMS, and the waste fee may possibly impact waste transportation and recycling, as shown in Appendix A Table A2. Thus, we used a two-step method to analyze the consistent impacts of the situations and institutions of SWMS on satisfaction using Equations (1) and (2), and the results are basically consistent.

#### 3.3.1. Determinants of Satisfaction with Community SWMS

Similar to the description statistics in Table 2, we found that community satisfaction varied based on the situations and institutions of SWMS in different regions and was not varied in different cities in Models 1–6, Table 4. When only analyzing the socioeconomic characteristics, environmental perceptions and community institutions, we discovered that when the possibility of living in the urban regions increased by one percentage point, the community satisfaction increased by 33.3%, as shown by Model 1 in Table 4. However, regarding the pilot cities and non-pilot cities, there was no discrepancy found in Model 5. After adding the situation of SWMS into the Ologit model, the results showed that that there were no significant differences in satisfaction between rural and urban regions in Model 2, which is not the same as the description result in Table 2. The possible reason for this is the gaps in the situations and institutions of SWMS among regions and cities, as shown in Table 2, and we also verified these results in Table 3 again.

Regarding the status of waste management services category, the results for the total sample in Model 2 of Table 4 showed that, except for waste transportation services, waste separation facilities and collection behavior and recycling services all significantly increased community satisfaction. When a resident reported that the possibility of providing waste management services (waste separation facilities and behavior, waste recycling services) increased by 1%, the levels of satisfaction increased by 52.6%, 39.7% and 77.6%, respectively (Model 2). In rural regions, only waste recycling services had a significant positive impact on satisfaction in Model 4. Community satisfaction increased by 56.3% when the availability of waste recycling services increased by 1%. In urban regions, all SWMS (waste separation, waste separation behavior, transportation and waste recycling services) had a significant positive impact on satisfaction, which increased by 74.8%, 63.3%, 72.6% and 95.9%, respectively, when the percentage of services increased by 1% (Table 4, Model 6).

With regards to the institutional nature of SWMS, waste management rules were the main factor affecting the community satisfaction rating in rural regions, which did not change after adding the situation of SWMS in Model 4, which was similar to the descriptive statistics. However, in the total and urban regions, the waste fee was the main factor. There was a varied result for the different samples after adding the situation of SWMS in Models 2, 4 and 6 of Table 4. In Model 1, waste advertisements and waste rules significantly affected the level of satisfaction. However, when adding the situation of SWMS in Models 2 and 6, the impacts of waste advertisements and rules were missing, and the waste management fee significantly decreased satisfaction in the total sample and in urban regions, at 35.8% and 40.1%, respectively (Models 2 and 6). One possible reason for this is that the waste fee is the source of funds for improving the situation and quality of SWMS. When the community asks the residents to submit a waste management fee, the community has a greater possibility to provide waste rules and advertisements. In addition, the residents have higher requirements for SWMS, and if the service does not meet the demands and expectations of the communities, their satisfaction would reduce.

In terms of the socioeconomic characteristics of the residents, the health of the residents had a significant impact on community satisfaction rating at the 0.001 level. When health increased by one level, satisfaction increased by 39.4%, 44.7% and 35.0% for the total, rural and urban samples, respectively (Models 2, 4 and 6 of Columns 3, 5 and 7, Table 4). In rural regions, the older the residents, the higher the community satisfaction. A higher income led to a significant decrease in community satisfaction, but gender and education and environmental activities did not lead to a change in satisfaction. In total and urban samples, we found that, except for health, other socioeconomic characteristics had no significant effect on the satisfaction in urban regions in Model 6. In addition, from Models 1–6, we found that environmental knowledge about solid waste environmental law and the pilot city lists in terms of waste separation and environmental activity had no impact on satisfaction with community waste solid management services, which was the same as the description results in Table 2.

#### 3.3.2. Determinants of National Satisfaction with SWMS

With the differences in the comparative analysis in Table 3, national satisfaction levels with SWMS between urban and rural areas were not significantly different; the situation for non-pilot and pilot cities is also the same. Models 7–12 of Table 4 also showed that national satisfaction was caused by varied factors in different regions and cities, and the result is comparable with the description statistics in Table 3.

With regards to the status of SWMS, waste recycling services were the only variable that affected satisfaction with national waste management services, and it only affected the satisfaction ratings in the total and urban samples, which is different from the results suggested by the descriptive statistics in Table 2. The possible reason for this is that recycling services provided by the local government are one part of SWMS and the last step of waste management, which decides the pollution level caused by waste. When a resident reported that the possibility of providing waste recycling services increased by 1% using total sample and urban sample data, satisfaction increased by 29.2% and 34.4%, respectively (Models 8 and 12). In rural regions, Model 10 showed that all waste management services had no significant impacts on the satisfaction with national SWMS.

In the waste services institutional policy category, the introduction of waste rules significantly improved satisfaction with national waste services in any samples; no impacts changed after adding the situation of SWMS in Models 8, 10 and 12, which is similar to the descriptive results in Table 2. When a resident reported that the possibility of making waste rules increased by 1% in the total, rural and urban samples, satisfaction increased by 52.7%, 62.2% and 49.6%, respectively (Models 8, 10 and 12). However, the waste management fee had no impact on national satisfaction, which is different from the results for community satisfaction in Models 1–6. These results implied the different impacts of central–local satisfaction with SWMS. However, waste-related advertising increased national satisfaction by 24.9% and 25.4% in the total and urban samples in Models 8 and 12. The possible reason for this is that waste advertising, as one part of the community work, showed the meaning and methods of waste separation in the community. When residents obtained more waste advertising services about waste separation from communities, their knowledge about community and national services became higher, and so their satisfaction with SWMS was increased.

In the socioeconomic characteristics of residents category, health was positively related to national satisfaction with SWMS, but education and income were negatively related to satisfaction. When residents’ health increased by one level, satisfaction increased by 27.7% and 23.7% for the total and urban samples, respectively (Table 4, Models 8, 10 and 12). Higher-educated and wealthy residents had a lower satisfaction with national waste management services. When the residents’ education increased one level, the satisfaction decreased by 19.5% only in the total sample from Model 8. When the residents’ income increased one level, the national satisfaction decreased by 26.4%, 35.9% and 25%, respectively. In rural regions, age increased satisfaction by 20.6%. In contrast to the satisfaction results for community solid waste management services, environmental knowledge about solid waste environmental law increased satisfaction with national waste management services in any sample. When the possibilities of knowing the waste environmental law increased by 1%, the satisfaction increased by 42.4%, 46% and 41.6% in Models 8, 10 and 12, respectively.

### 3.4. Robustness Test

To enhance the reliability of the results, we performed a robustness test using the Oprobit and OLS regression model, and the results are shown in Appendix A Table A3. The results are basically consistent with Table 4, so the results of the analysis above were considered to be moderately robust.

## 4. Discussion

This paper compared the difference between central and local satisfaction using community and national satisfaction with SWMS and showed their determinants. Overall, the satisfaction with community SWMS in the total sample is similar with national SWMS, but the satisfaction with community SWMS in rural regions is lower than national SWMS, and the situation is contrary in the urban regions. The result in rural regions is the same as precious studies about environmental services, medical services and public services, showing that the satisfaction with policy implementers is lower than that with policy makers based on the strong relationship between government trust and satisfaction [82,83,84]. However, the result in urban regions is different, and the reason may be the sufficient degree of waste management services in the communities of urban regions [41,42,81,85]. The results were the same as the results in Western countries. When comparing between rural and urban regions, we found that the satisfaction levels of different residents varied, and community satisfaction in urban regions is higher than rural regions. The reason is the dualism of urban and rural regions and the different approaches and concentration ratios of services. The situation regarding national satisfaction is contrary, which is that rural residents’ satisfaction is higher than urban residents, which is consistent with satisfaction with public services in previous studies [29,30,31,32,33,34,35,54,55,56].

Concerning the community satisfaction with SWMS, the most important determinant is the status of SWMS, especially in the urban regions. Hence, community waste services are very important, and the higher the possibility of providing SWMS, the higher the community satisfaction in the total sample and urban sample. However, in urban regions, the waste fee decreased the community satisfaction. In rural regions, only waste recycling services and waste rules mainly affected the satisfaction. The possible explanations include two aspects. On the one hand, in terms of the process of making policy, the basic work for local government or communities is to provide public services with flexible implementation methods that are adapted to social and economic development levels below the mandatory or exemplary policy [63,64,65]. The government documents or regulations of 60 areas requested the residents to distribute the waste into two categories, three categories or five categories, while only 30% separate waste into four categories, which is the same as the national waste separation standards [78]. Thus, the residents have the chance to engage in free-riding and have no any punishment in most cities [16,86]. Compared with other public services such as health or education services, solid waste services are pure public goods and basic environmental services, but not very important services, so the demand or expectation may be general and the services found to be generally equal—a situation that is basically the same in the different social classes [65,68,87]. On the other hand, the situations and qualities of community waste management services provided by the local government can be experienced by the residents, and the distance is not great from the residents’ living spaces, so the residents can give an accurate evaluation of the SWMS [88]. Although the situation and quality of waste management services is different in regions and cities and the situation and quality of SWMS in rural regions and non-pilot cities is lower than the pilot cities, they are better than in the past from the time perspective. In rural regions, despite comprehensive waste management services still being lacking, the waste services almost having not charged and the willingness of paying for waste services being low, “village collection, town transportation, county disposal” has become the main mode of service under the support of funds from rural environmental improvement projects, and the living environment and waste management have been improved to a great extent [37,79,80]. In urban regions, the status of waste management services is different between non-pilot and pilot cities in terms of national mandatory waste separation policy. Pilot cities such as Shanghai and Shenzhen, have formulated a basic waste management system: waste separation at home, waste collection in the community, transportation and treatment of local government with adequate financial support from special bonds or public–private partnership, relatively good payment and management institutions and effective public environmental education systems such as waste separation advertising boards in public places, the dandelion project for training lecturers, a waste separation science museum and interesting waste separation-promoting activities organized by firms and NGO [8,9,10,51,89,90,91]. In non-pilot cities, because of capital shortage for investing in SWMS, local governments have generally chosen some communities as pilot areas and installed waste separation facilities, sent the guidelines of waste separation, built and organized volunteer teams, improved the intentions of waste separation with the methods of credit exchange and the free sending of daily goods and optimized the waste management systems with limited public funds and resources [49,78,92,93,94].

Regarding the national satisfaction with SWMS, the main factor is the community rules of SWMS. This was verified by other previous studies [62] and practices in the process of making and implementing policy [63,66]. In the urban regions, recycling services and waste management fees can also increase the national satisfaction. The possible explanations for this include two aspects. On the one hand, from the process of making policy, the institutions were the first and most efficient step in the cost and benefit analysis, gradually improving the level and qualities of services. In practice, making community rules was also the first step for implementing government policy, and then advertising rules and activities can add knowledge of how to separate waste. On the other hand, the central government is located in the capital cities, which is far away from the local communities, and their policies were told through the media or advertisements, so their services were not easily evaluated by the residents [69]. Thus, the results suggest that the rules can impact the national satisfaction, and the mandatory waste management policy did not reduce the national satisfaction. Thus, through the differences in culture and institutions, mandatory national policies and flexible local services can also be applied to other countries because of the public properties of waste services and the residents’ universal demands for a beautiful life environment and sustainable development [14,54,95].

Concerning socioeconomic characteristics and environmental perception, health is the most important factor for community and national satisfaction—the reason may be that a healthy resident has greater wishes to change the dirty and messy environment. In additions, the residents’ health condition is also a very important factor that increased satisfaction for all samples, because healthy residents can use the waste management services, including separating waste, throwing waste into the waste separation facilities and learning the waste transportation and disposal services [8,96]. Income and education decreased the national satisfaction with SWMS and have no impacts on community satisfaction, which is the same as the results of precious studies [22].

Although this paper gives a new idea about how to make a comparison of the central–local satisfaction with SWMS, limitations of this paper also exist and include four points. First, the paper is simply exploratory research about satisfaction with SWMS in China, and the result only affects the general situation and is not representative for the overall situation in China, because the sample is not randomly selected and not a nationally representative sample. Second, the result simply shows the correlational relationship between SWMS and satisfaction and does not give the precise causal relationships regarding the effect of satisfaction with SWMS. Third, the study may have some selection and reporting bias. Although the sample included 185 cities in China, we used the seed investigators to send the questionnaires and did not randomly select the sample, so there was some selection bias in the sample. In the questions regarding the situation of SWMS, some residents did not know the answers for the SWMS, so there was some reporting bias due to the lack of residents’ knowledge about local waste management services. Fourth, the results of this paper are based on the data in China, so we cannot precisely compare the result to other countries, though the results may be adaptable to other countries because of the characteristics of public services.

## 5. Conclusions and Suggestions

The results showed that the differences in central–local satisfaction with SWMS in the total sample were almost non-existent, but in the different regions, the results were varied. The community satisfaction with solid waste management services in urban regions was greater than national services or policy, but resident satisfaction with national waste services in rural regions was higher than community services. The main determinants of community satisfaction were waste management services and waste management fees, and the national satisfaction is impacted by waste recycling services and waste rules and advertisements. However, the factors affecting community and national satisfaction differed depending on the sample type. In rural regions, waste recycling services and waste rules affected community services satisfaction, whereas only waste rules affected satisfaction with national waste services. In urban regions, all waste management services and waste fees had significant impacts on satisfaction with community services, but only waste recycling services and waste advertisements and rules increased satisfaction with the national waste management services.

We propose several suggestions for improving waste management services, institutional policies, and systems. First, the local government or communities should continue to establish and invest in waste management facilities’ coverage in rural and urban areas. They should also strengthen the supply of comprehensive waste management services, especially the supply of waste treatment services and encourage the recycling and reuse of waste and thus improve satisfaction at the community and national levels. In urban regions, the integrality of waste management services in pilot and non-pilot cities should be constantly improved, which will increase waste management capacity and efficiency. Second, with regards to institutions, the government should at first increase the establishment of waste management institutions in communities across rural and urban regions, which will increase satisfaction with community and national waste services and should improve their performance. At the same time, the government should increase its advertising efforts and charge reasonably priced waste fees for better waste management services. Third, especially in the aging society, some facilities or services that are convenient for less healthy residents could be considered in waste management systems to optimize waste management systems and services, which should increase satisfaction. For example, the government could add a number of waste separation facilities and design a lower trash can. Fourth, the local government or community should increasingly advertise the waste management systems and deepen the knowledge of waste, not only guiding how to separate waste but also leading the community residents to visit the waste transportation and disposal services.

In the future, studies should be developed and directed in terms of the following four aspects. The first is continuing to explore the impact of the quality of waste services on satisfaction in the different regions, which can steadily improve the performance or satisfaction with waste management services. Second is performing a comprehensive survey about the knowledge and utilization of waste management services in China; then, some intervention methods can be designed to increase the knowledge level of services and institutions of residents and increase the waste separation behavior. Third is to enhance the studies about new advertising methods about waste information or institutions and services targeting different residents, especially older people, such as “Internet + Time”, new media, short videos and regularly disseminating the knowledge daily. Fourth is comparing the similarities and differences of SWMS policies and services among different countries because of the different cultures and institutions and comparing the satisfaction with different public services, which may vary with the characters, subjects and patterns of providers.

## Figures and Tables

**Table 1:** Descriptive statistics.

Variables	Definition	Count	Mean	SD
Socioeconomic characteristics				
Gender	(0) Male; (1) Female.	1189	0.57	0.49
Age	(1) Lower than 24; (2) ≥25 and ≤34; (3) ≥35 and ≤44; (4) ≥45 and ≤54; (5) More than 55.	1189	1.71	1.12
Education	(1) Junior high school or below; (2) Senior high school; (3) Bachelor’s degree; (4) Master’s degree or higher.	1189	2.95	0.64
Health	(1) Not good; (2) General; (3) Good; (4) Very good.	1189	2.93	0.83
Income per month (USD)	(1) Lower than 782; (2) 782 to 1564; (3) 1564 to 2346; (4) More than 2346.	1189	1.92	1.03
Environmental perception				
Environment activity	(0) No; (1) Yes.	1189	0.30	0.46
Environmental law	(0) No; (1) Known; (2) Very well known.	1189	0.74	0.72
City list ^1^	(0) No; (1) Yes.	1189	0.86	0.35
Status of SWMS in the community				
Waste separation facilities	(0) No; (1) Yes	1189	0.28	0.45
Waste separation behavior ^2^	(0) No; (1) Yes.	1183	0.62	0.49
Waste transportation ^3^	(0) No; (1) Yes.	746	0.87	0.34
Waste recycling	(0) No; (1) Yes.	1103	0.59	0.49
Institution of SWMS in the community				
Waste advertisement	(0) No; (1) Yes.	1189	0.64	0.48
Waste rules	(0) No; (1) Yes.	1189	0.56	0.50
Waste fee	(0) No; (1) Yes.	997	0.53	0.50
Location				
Regions	(0) Rural; (1) Urban	1189	0.70	0.46
Pilot city ^4^	(0) Non-pilot; (1) Pilot.	835	0.49	0.50
Satisfaction of SWMS				
Community Waste satisfaction	(1) Very unsatisfied;(2) Unsatisfied; (3) Neutral; (4) Satisfied; (5) Very satisfied.	1189	3.18	0.90
National Waste satisfaction	(1) Very unsatisfied;(2) Unsatisfied; (3) Neutral; (4) Satisfied; (5) Very satisfied.	1189	3.15	0.90

Note: ^1^ City list shows whether residents know the pilot city list. ^2^ Waste separation behavior includes waste separation collection workers separating waste in the community and residents separating waste in the house. ^3^ In the waste transportation, 37% of residents did not know the situation of waste transportation. ^4^ Pilot city shows whether the residents lived in a waste separation pilot city, which was calculated based on the filled-in address and national documents in China.

**Table 2:** Solid waste management services and institutions across regions and cities in China.

Variables	Value	Total		Rural ^2^		Urban ^2^		Rural and Urban ^3^	Non-Pilot City ^2^		Pilot City ^2^		Non-Pilot and Pilot City ^3^
		Num ^1^	Mean	Num	Mean	Num	Mean		Num	Mean	Num	Mean	
Status of SWMS													
Waste separation facilities	No	853	0.72	297	0.84	556	0.67		342	0.80	214	0.52	
	Yes	336	0.28	57	0.16	279	0.33	−0.17 ***	84	0.20	195	0.48	−0.28 ***
Waste separation	No	452	0.38	155	0.44	297	0.36		179	0.42	118	0.29	
	Yes	731	0.62	196	0.56	535	0.64	−0.08 **	246	0.58	289	0.71	−0.13 ***
Waste Transportation ^4^	No	98	0.13	39	0.17	59	0.11		27	0.10	32	0.13	
	Yes	648	0.87	193	0.83	455	0.89	−0.05 *	235	0.90	220	0.87	0.02
Waste recycling	No	456	0.41	159	0.48	297	0.39		164	0.42	133	0.35	
	Yes	647	0.59	174	0.52	473	0.61	−0.09 **	224	0.58	249	0.65	−0.07 *
Institutions of SWMS													
Waste advertisement	No	429	0.36	149	0.42	280	0.34		189	0.44	91	0.22	
	Yes	760	0.64	205	0.58	555	0.66	−0.08 **	237	0.56	318	0.78	−0.22 ***
Waste Rule	No	521	0.44	166	0.47	355	0.43		232	0.54	123	0.30	
	Yes	668	0.56	188	0.53	480	0.57	−0.04	194	0.46	286	0.70	−0.24 ***
Waste fee	No	465	0.47	226	0.76	239	0.34		107	0.30	132	0.39	
	Yes	532	0.53	72	0.24	460	0.66	−0.42 ***	250	0.70	210	0.61	0.09 *

Note: ^1^ Num is the sample number. ^2^ Rural is the sample in rural regions. Urban is the sample in urban regions. Non-pilot and pilot city are the same. ^3^ This shows the difference between rural and urban regions and non-pilot and pilot cities. ^4^ The sample is lower than other variables, as many residents did not know whether the community had transportation services. *** *p* < 0.01, ** *p* < 0.05, * *p* < 0.1.

**Table 3:** Solid waste management services and satisfaction with community and national SWMS among rural and urban regions in China.

Variables	Value	Total			Rural			Urban		
		Number	Satisfaction		Number	Satisfaction		Number	Satisfaction	
			Community	Nation		Community	Nation		Community	Nation
Mean ^1^		1189	3.18	3.15	354	3.06	3.24	835	3.23	3.11
Status of SWMS										
Waste separation	No	853	3.09	3.13	297	3.03	3.22	556	3.13	3.09
facilities	Yes	336	3.39	3.18	57	3.19	3.35	279	3.43	3.15
	Dif ^2^		−0.30 ***	−0.05		−0.16	−0.13		−0.30 ***	−0.06
Waste separation	No	452	2.98	3.08	155	2.89	3.12	297	3.03	3.07
behavior	Yes	731	3.30	3.18	196	3.19	3.34	535	3.34	3.13
	Dif		−0.32 ***	−0.10		−0.30 **	−0.22 *		−0.32 ***	−0.06
Waste	No	98	3.05	2.94	39	3.23	3.05	59	2.93	2.86
transportation	Yes	648	3.25	3.21	193	3.11	3.27	455	3.31	3.18
	Dif		−0.20	−0.27 **		0.12	−0.22		−0.37 **	−0.32 *
Waste	No	456	2.91	3.03	159	2.84	3.16	297	2.95	2.97
recycling	Yes	647	3.37	3.25	174	3.28	3.34	473	3.40	3.22
	Dif		−0.46 ***	−0.22 ***		−0.44 ***	−0.18		−0.45 ***	−0.25 ***
Institutions of SWMS										
Waste	No	429	2.94	3.03	149	2.89	3.10	280	2.96	2.99
advertisement	Yes	760	3.32	3.21	205	3.18	3.35	555	3.37	3.17
	Dif		−0.38 ***	−0.19 ***		−0.30 **	−0.25 *		−0.40 ***	−0.18 **
Waste rules	No	521	3.00	2.99	166	2.81	3.04	355	3.09	2.97
	Yes	668	3.32	3.27	188	3.28	3.43	480	3.34	3.20
	Dif		−0.32 ***	−0.27 ***		−0.47 ***	−0.39 ***		−0.25 ***	−0.23 ***
Waste fee	No	465	3.19	3.23	226	3.08	3.28	239	3.31	3.19
	Yes	532	3.14	3.11	72	3.06	3.18	460	3.16	3.10
	Dif		0.05	0.13 *		0.02	0.10		0.15 *	0.09

Note: ^1^ Mean is the mean of community and national satisfaction level in the total, rural and urban sample. ^2^ Dif is the difference between two groups. *** *p* < 0.01, ** *p* < 0.05, * *p* < 0.1.

**Table 4:** Determinants of satisfaction with communities and national SWMS in China.

Independent Variables	Community Waste Satisfaction						National Waste Satisfaction					
Sample	Total		Rural		Urban		Total		Rural		Urban	
Model	(1)	(2)	(3)	(4)	(5)	(6)	(7)	(8)	(9)	(10)	(11)	(12)
Regions	0.333 **	0.144					−0.159	−0.189				
	(0.164)	(0.216)					(0.172)	(0.160)				
Pilot city					−0.089	−0.197					−0.094	−0.044
					(0.177)	(0.210)					(0.223)	(0.200)
Gender	−0.105	−0.015	−0.286	−0.172	−0.018	0.037	0.154	0.059	0.257	0.221	0.122	−0.003
	(0.139)	(0.141)	(0.244)	(0.268)	(0.183)	(0.197)	(0.144)	(0.154)	(0.232)	(0.275)	(0.150)	(0.167)
Age	−0.080	0.072	0.340 **	0.384 ***	−0.157 **	0.024	0.047	0.101	−0.004	0.206 *	0.056	0.070
	(0.065)	(0.061)	(0.137)	(0.143)	(0.062)	(0.072)	(0.051)	(0.069)	(0.108)	(0.121)	(0.057)	(0.080)
Edu	−0.118	0.122	0.007	0.186	−0.054	0.174	−0.325 ***	−0.195 *	−0.412 ***	−0.116	−0.294 **	−0.213
	(0.115)	(0.134)	(0.159)	(0.200)	(0.120)	(0.151)	(0.100)	(0.103)	(0.149)	(0.164)	(0.146)	(0.139)
Health	0.404 ***	0.394 ***	0.346 ***	0.447 ***	0.447 ***	0.350 ***	0.285 ***	0.277 ***	0.366 **	0.337	0.239 ***	0.237 **
	(0.068)	(0.099)	(0.116)	(0.155)	(0.085)	(0.113)	(0.079)	(0.092)	(0.175)	(0.232)	(0.088)	(0.114)
Income	0.044	−0.038	−0.313 ***	−0.311 **	0.130 **	0.060	−0.215 ***	−0.264 ***	−0.299 **	−0.359 **	−0.189 **	−0.250 ***
	(0.057)	(0.079)	(0.118)	(0.146)	(0.064)	(0.087)	(0.065)	(0.077)	(0.150)	(0.177)	(0.079)	(0.091)
Environment activity	0.180	0.071	0.331	0.462	0.155	−0.088	0.048	−0.055	0.044	0.233	0.045	−0.187
	(0.120)	(0.146)	(0.291)	(0.357)	(0.148)	(0.205)	(0.129)	(0.153)	(0.252)	(0.321)	(0.169)	(0.179)
Environmental law	−0.022	−0.014	0.186	0.275	−0.092	−0.060	0.388 ***	0.424 ***	0.360 **	0.460 *	0.395***	0.416 ***
	(0.171)	(0.178)	(0.290)	(0.331)	(0.141)	(0.153)	(0.103)	(0.155)	(0.181)	(0.239)	(0.095)	(0.144)
City list	0.138	0.352	0.229	0.150	0.053	0.446	0.356 *	0.204	0.683 ***	0.553	0.196	−0.003
	(0.172)	(0.277)	(0.312)	(0.411)	(0.213)	(0.371)	(0.185)	(0.207)	(0.258)	(0.362)	(0.225)	(0.268)
Waste advertisement	0.611 ***	0.058	0.088	−0.314	0.876 ***	0.211	0.171	0.249 *	0.132	0.166	0.199	0.254 *
	(0.120)	(0.146)	(0.243)	(0.277)	(0.155)	(0.174)	(0.139)	(0.143)	(0.283)	(0.313)	(0.159)	(0.152)
Waste rules	0.451 ***	0.209	0.817 ***	0.715 ***	0.313 **	−0.119	0.465 ***	0.527 ***	0.739 ***	0.622 **	0.359 **	0.496 *
	(0.141)	(0.192)	(0.256)	(0.247)	(0.145)	(0.228)	(0.122)	(0.183)	(0.249)	(0.296)	(0.152)	(0.253)
Waste Fee	−0.129	−0.358 **	−0.139	−0.400	−0.170	−0.401 **	−0.160	−0.160	−0.127	−0.325	−0.194	−0.147
	(0.147)	(0.182)	(0.299)	(0.406)	(0.156)	(0.190)	(0.121)	(0.147)	(0.269)	(0.287)	(0.149)	(0.202)
Waste separation facilities		0.526**		−0.040		0.748 ***		−0.052		0.060		−0.044
		(0.212)		(0.642)		(0.164)		(0.256)		(0.339)		(0.281)
Waste separation behavior		0.397 **		0.091		0.633 ***		−0.105		0.080		−0.250
		(0.174)		(0.414)		(0.175)		(0.157)		(0.304)		(0.188)
Waste transportation		0.346		−0.279		0.726 **		0.406		0.399		0.431
		(0.225)		(0.376)		(0.357)		(0.294)		(0.302)		(0.448)
Waste recycling		0.776 ***		0.563 **		0.959 ***		0.292 *		0.191		0.344 *
		(0.170)		(0.278)		(0.227)		(0.156)		(0.242)		(0.198)
Observations	997	642	298	200	699	442	997	642	298	200	699	442

Note: Robust standard errors in parentheses. *** *p* < 0.01, ** *p* < 0.05, * *p* < 0.1.

## Data Availability

The data presented in this study are available on request from the corresponding authors.

## Appendix A

**Table A1:** Solid waste management services and satisfaction with community and national SWMS among non-pilot and pilot cities in urban China.

Variables	Value	Urban Regions			Non-Pilot City			Pilot City		
		Number	Waste Satisfaction		Number	Waste Satisfaction		Number	Waste Satisfaction	
			Community	Nation		Community	Nation		Community	Nation
Mean ^1^		835	3.23	3.11	426	3.19	3.10	409	3.27	3.11
Status of SWMS										
Waste separation facilities	No	556	3.13	3.09	342	3.14	3.10	214	3.12	3.07
	Yes	279	3.43	3.15	84	3.42	3.12	195	3.44	3.16
	Dif ^2^		−0.30 ***	−0.06		−0.28 **	−0.02		−0.32 ***	−0.09
Waste separation behavior	No	297	3.03	3.07	179	3.01	3.04	118	3.06	3.11
	Yes	535	3.34	3.13	246	3.33	3.14	289	3.36	3.11
	Dif		−0.32 ***	−0.06		−0.32 ***	−0.10		−0.30 **	0.00
Waste transportation	No	59	2.93	2.86	27	2.93	2.78	32	2.94	2.94
	Yes	455	3.31	3.18	235	3.25	3.19	220	3.36	3.17
	Dif		−0.38 **	−0.32 *		−0.33	−0.41 *		−0.42 *	−0.24
Waste recycling	No	297	2.95	2.97	164	2.93	2.98	133	2.97	2.95
	Yes	473	3.40	3.22	224	3.36	3.21	249	3.44	3.22
	Dif		−0.45 ***	−0.25 ***		−0.43 ***	−0.23 **		−0.47 ***	−0.27 **
Institutions of SWMS										
Waste advertisement	No	355	3.09	2.97	232	3.06	2.98	123	3.15	2.97
	Yes	480	3.34	3.20	194	3.35	3.25	286	3.33	3.17
	Dif		−0.25 ***	−0.23 ***		−0.29 ***	−0.27 ***		−0.18	−0.20 *
Waste rules	No	280	2.96	2.99	189	2.95	2.95	91	2.99	3.07
	Yes	555	3.37	3.17	237	3.38	3.22	318	3.35	3.13
	Dif		−0.41 ***	−0.18 **		−0.43 ***	−0.27 ***		−0.36 ***	−0.06
Waste fee	No	239	3.31	3.19	107	3.18	3.18	132	3.41	3.20
	Yes	460	3.16	3.10	250	3.20	3.10	210	3.11	3.09
	Dif		0.15 *	0.09		−0.02	0.07		0.23 **	0.11

Note: ^1^ Mean is the mean of community and national satisfaction level in the total, rural and urban sample. ^2^ Dif is the difference between two groups. *** *p* < 0.01, ** *p* < 0.05, * *p* < 0.1.

**Table A2:** The relationship between socioeconomics and environmental knowledge and community institution variables and SWMS.

Variables	Total				Rural				Urban			
	Separation Facilities	Separation Behavior	Transportation	Waste Recycling	Separation Facilities	Separation Behavior	Transportation	Waste Recycling	Separation Facilities	Separation Behavior	Transportation	Waste Recycling
Model	(1)	(2)	(3)	(4)	(5)	(6)	(7)	(8)	(9)	(10)	(11)	(12)
Regions	0.494 ***	0.215 **	−0.031	0.337 ***								
	(0.147)	(0.092)	(0.146)	(0.091)								
Pilot city									0.694 ***	0.160 *	−0.179	−0.061
									(0.182)	(0.093)	(0.184)	(0.103)
Gender	−0.059	−0.029	0.121	0.124	−0.283	−0.165	−0.079	0.131	−0.108	0.002	0.236	0.123
	(0.101)	(0.079)	(0.159)	(0.081)	(0.225)	(0.126)	(0.188)	(0.166)	(0.127)	(0.094)	(0.209)	(0.106)
Age	−0.004	−0.049	0.105 **	−0.024	0.007	0.071	0.152	0.037	−0.018	−0.061	0.108 *	−0.042
	(0.046)	(0.051)	(0.052)	(0.030)	(0.115)	(0.104)	(0.153)	(0.075)	(0.043)	(0.041)	(0.065)	(0.037)
Edu	−0.052	0.031	−0.213 **	−0.197 ***	−0.284	0.134	−0.220	−0.241	0.031	0.009	−0.152	−0.171 **
	(0.128)	(0.051)	(0.084)	(0.068)	(0.210)	(0.114)	(0.137)	(0.147)	(0.091)	(0.054)	(0.109)	(0.069)
Health	0.036	0.040	0.162 **	0.030	−0.030	0.071	−0.079	0.061	0.118	0.036	0.289 ***	0.028
	(0.079)	(0.058)	(0.076)	(0.048)	(0.119)	(0.099)	(0.149)	(0.085)	(0.091)	(0.064)	(0.096)	(0.061)
Income	0.026	0.005	0.066	0.070	0.043	−0.000	0.281 *	0.038	−0.024	−0.017	0.023	0.069
	(0.052)	(0.050)	(0.050)	(0.046)	(0.118)	(0.082)	(0.170)	(0.081)	(0.056)	(0.059)	(0.060)	(0.051)
Environment activity	0.120	0.012	0.164	−0.112	−0.091	0.247	0.175	−0.193	0.174	−0.116	0.177	−0.076
	(0.100)	(0.106)	(0.117)	(0.085)	(0.225)	(0.219)	(0.202)	(0.140)	(0.116)	(0.100)	(0.162)	(0.129)
Environmental law	0.044	0.240 ***	−0.061	0.069	0.597 ***	0.433 ***	0.100	0.186 *	−0.121	0.168 **	−0.164	0.011
	(0.093)	(0.065)	(0.081)	(0.054)	(0.139)	(0.145)	(0.133)	(0.099)	(0.094)	(0.069)	(0.108)	(0.064)
City list	−0.029	−0.237 **	0.383	0.013	−0.363 *	−0.250	0.180	0.241	0.036	−0.314 **	0.450	−0.117
	(0.155)	(0.101)	(0.234)	(0.102)	(0.205)	(0.187)	(0.257)	(0.177)	(0.205)	(0.151)	(0.283)	(0.135)
Waste advertisement	0.467 ***	0.671 ***	0.008	0.192 **	0.184	0.639 ***	0.015	0.241	0.533 ***	0.670 ***	0.055	0.176 *
	(0.113)	(0.083)	(0.143)	(0.091)	(0.233)	(0.143)	(0.214)	(0.207)	(0.141)	(0.105)	(0.192)	(0.091)
Waste rules	0.738 ***	0.631 ***	0.232 **	0.294 ***	0.564 ***	0.543 **	0.332 *	0.087	0.700 ***	0.647 ***	0.253 *	0.389 ***
	(0.104)	(0.116)	(0.118)	(0.114)	(0.185)	(0.213)	(0.183)	(0.176)	(0.114)	(0.111)	(0.146)	(0.136)
Waste Fee	0.064	−0.032	0.233*	−0.245 **	−0.004	0.014	0.531 *	−0.031	0.112	−0.055	0.117	−0.314 ***
	(0.100)	(0.081)	(0.133)	(0.096)	(0.228)	(0.178)	(0.275)	(0.158)	(0.109)	(0.077)	(0.203)	(0.122)
Observations	997	995	682	932	298	296	213	282	699	699	469	650

Note: Robust standard errors in parentheses. *** *p* < 0.01, ** *p* < 0.05, * *p* < 0.1.

**Table A3:** Satisfaction with communities and national solid waste management services in China.

Independent Variables	Community Waste Satisfaction						Nation Waste Satisfaction					
Sample	Total		Rural		Urban		Total		Rural		Urban	
Model	Oprobit	OLS	Oprobit	OLS	Oprobit	OLS	Oprobit	OLS	Oprobit	OLS	Oprobit	OLS
	(1)	(2)	(3)	(4)	(5)	(6)	(7)	(8)	(9)	(10)	(11)	(12)
Place of residents	0.086	0.076					−0.064	−0.038				
	(0.111)	(0.092)					(0.091)	(0.077)				
Pilot Waste separation city					−0.126	−0.114					−0.006	−0.008
					(0.117)	(0.092)					(0.116)	(0.097)
Gender	−0.008	0.008	−0.116	−0.092	0.037	0.046	0.048	0.044	0.113	0.103	0.020	0.020
	(0.073)	(0.059)	(0.150)	(0.132)	(0.099)	(0.077)	(0.083)	(0.071)	(0.160)	(0.149)	(0.093)	(0.080)
Age	0.036	0.034	0.237 ***	0.208 ***	0.004	0.006	0.042	0.024	0.104 *	0.075	0.024	0.010
	(0.036)	(0.032)	(0.075)	(0.069)	(0.042)	(0.034)	(0.037)	(0.029)	(0.063)	(0.056)	(0.044)	(0.035)
Edu	0.087	0.085	0.174 *	0.175 *	0.089	0.076	−0.099 *	−0.079	−0.046	−0.024	−0.109	−0.088
	(0.072)	(0.060)	(0.103)	(0.089)	(0.082)	(0.066)	(0.057)	(0.049)	(0.094)	(0.088)	(0.082)	(0.069)
Health	0.209 ***	0.157 ***	0.240 ***	0.184 **	0.177 ***	0.120 **	0.141 ***	0.109 **	0.149	0.115	0.130 **	0.097 *
	(0.054)	(0.043)	(0.083)	(0.072)	(0.061)	(0.047)	(0.053)	(0.046)	(0.126)	(0.111)	(0.065)	(0.056)
Income	−0.013	−0.016	−0.197 ***	−0.174 **	0.056	0.042	−0.145 ***	−0.119 ***	−0.182 *	−0.139	−0.142 ***	−0.116 ***
	(0.042)	(0.036)	(0.076)	(0.070)	(0.046)	(0.037)	(0.043)	(0.037)	(0.104)	(0.095)	(0.050)	(0.042)
Environment activity	0.069	0.049	0.274	0.215	−0.022	−0.022	−0.002	−0.009	0.125	0.084	−0.065	−0.054
	(0.081)	(0.068)	(0.188)	(0.157)	(0.133)	(0.116)	(0.085)	(0.071)	(0.164)	(0.144)	(0.100)	(0.080)
Environemtal law	−0.045	−0.070	0.123	0.067	−0.071	−0.083	0.192 **	0.139 **	0.218	0.156	0.189 ***	0.136**
	(0.090)	(0.073)	(0.173)	(0.147)	(0.077)	(0.059)	(0.076)	(0.067)	(0.138)	(0.132)	(0.066)	(0.054)
City list	0.200	0.181	0.116	0.103	0.262	0.221	0.103	0.087	0.262	0.234	0.000	−0.005
	(0.152)	(0.134)	(0.234)	(0.216)	(0.197)	(0.162)	(0.117)	(0.105)	(0.197)	(0.182)	(0.153)	(0.134)
Waste separation facilities	0.256 **	0.184 *	0.031	−0.022	0.382 ***	0.286 ***	−0.053	−0.071	−0.004	−0.041	−0.050	−0.063
	(0.120)	(0.102)	(0.344)	(0.300)	(0.097)	(0.077)	(0.144)	(0.122)	(0.182)	(0.170)	(0.154)	(0.127)
Waste separation behavior	0.206 **	0.156 *	0.002	−0.003	0.354 ***	0.263 ***	−0.096	−0.091	0.057	0.029	−0.199 *	−0.163 *
	(0.100)	(0.080)	(0.223)	(0.191)	(0.103)	(0.074)	(0.092)	(0.077)	(0.167)	(0.149)	(0.102)	(0.086)
Waste transportation	0.134	0.106	−0.170	−0.147	0.333 *	0.260 *	0.224	0.197	0.175	0.147	0.261	0.228
	(0.122)	(0.100)	(0.200)	(0.175)	(0.196)	(0.155)	(0.154)	(0.135)	(0.160)	(0.150)	(0.225)	(0.188)
Waste recycling	0.472 ***	0.396 ***	0.360 **	0.300 **	0.566 ***	0.454 ***	0.189 **	0.167 **	0.114	0.106	0.223 **	0.188 **
	(0.095)	(0.086)	(0.154)	(0.147)	(0.124)	(0.095)	(0.087)	(0.076)	(0.139)	(0.134)	(0.108)	(0.092)
Waste advertisation	0.028	0.024	−0.191	−0.134	0.125	0.084	0.144*	0.118*	0.073	0.060	0.164 **	0.130 **
	(0.085)	(0.073)	(0.154)	(0.130)	(0.089)	(0.072)	(0.079)	(0.068)	(0.169)	(0.155)	(0.081)	(0.065)
Waste rules	0.140	0.115	0.423***	0.336***	−0.049	−0.029	0.300 ***	0.250 ***	0.344 **	0.303*	0.288 **	0.233 **
	(0.108)	(0.088)	(0.136)	(0.116)	(0.127)	(0.101)	(0.105)	(0.087)	(0.171)	(0.154)	(0.137)	(0.110)
Waste Fee	−0.201 *	−0.158 *	−0.245	−0.215	−0.208 **	−0.151 *	−0.075	−0.049	−0.187	−0.153	−0.051	−0.027
	(0.105)	(0.093)	(0.220)	(0.206)	(0.104)	(0.083)	(0.081)	(0.070)	(0.167)	(0.153)	(0.108)	(0.093)
Observations	642	642	200	200	442	442	642	642	200	200	442	442
R-squared ^1^	0.059	0.132	0.070	0.157	0.080	0.176	0.047	0.106	0.062	0.131	0.044	0.102

Note: ^1^ When the model is Oprobit, R-squared is Pseudo R-squared. Robust standard errors in parentheses. *** *p* < 0.01, ** *p* < 0.05, * *p* < 0.1.
